# A retrospective analysis of medication prescription records for determining the levels of compliance and persistence to urate-lowering therapy for the treatment of gout and hyperuricemia in The Netherlands

**DOI:** 10.1007/s10067-018-4127-x

**Published:** 2018-05-02

**Authors:** C. A. Janssen, M. A. H. Oude Voshaar, H. E. Vonkeman, M. Krol, M. A. F. J. van de Laar

**Affiliations:** 1 Department of Psychology, Health and Technology, University of Twente, PO BOX 217, 7500 AE Enschede, The Netherlands; 2 Arthritis Center Twente, Medisch Spectrum Twente, Enschede, The Netherlands; 3 IQVIA, Real World Evidence Solutions, Amsterdam, The Netherlands

**Keywords:** Compliance, Gout, Persistence, Urate-lowering therapy

## Abstract

Urate-lowering therapy (ULT) is a recommended life-long treatment for gout patients. However, despite these recommendations, recurrent gout attacks are commonly observed in clinical practice. The purpose of this study was to assess the levels of compliance and persistence to ULT in The Netherlands, in order to reflect on the current gout care delivered by health professionals. Anonymous prescription records were obtained from IQVIA’s Dutch retrospective longitudinal prescription database, containing ULT dispensing data for allopurinol, febuxostat, and benzbromarone from November 2013 to July 2017. Compliance to ULT was determined by calculating the proportion of days covered (PDC) over 12 months. Persistence over 12 months was evaluated by determining the time to discontinuation, without surpassing a refill gap of > 30 days. Association of PDC and persistence with age, gender, and first prescriber were examined using beta regression- and cox-regression models, respectively. There were 45,654 patients who met the inclusion criteria. Overall, 51.7% of the patients had a ULT coverage of ≥ 80% of the days in 1 year (PDC ≥ 0.80), and 42.7% of the patients were still persistent after 1 year. Men, older patients, and patients whose first prescriber was a rheumatologist were more persistent and had a higher PDC. Our results show that medication adherence to ULT after 1 year is suboptimal, considering that current guidelines recommend ULT as a life-long treatment. Future studies addressing the reasons for treatment cessation and improving treatment adherence seem warranted.

## Introduction

Gout, which has become increasingly prevalent over the last decades, is currently one of the most common forms of inflammatory arthritis [1, 2]. Acute gout flares result from prolonged hyperuricemia, which may lead to the formation and deposition of mono-sodium urate crystals in and around the joints, triggering an inflammatory response. Gout is associated with pain, swelling, tenderness, and erythema and may lead to physical disability and reduced quality of life [3, 4].

One of the major goals of gout management is lowering and managing serum urate levels by using urate-lowering therapy (ULT) to prevent gout attacks. Current guidelines recommend initiating ULT in patients with recurring gout flares and tophi, and to consider initiating ULT in all patients upon definite diagnosis of gout [5]. However, despite these recommendations and the availability of effective ULT, recurrent attacks of gouty arthritis are commonly observed in clinical practice [6]. These may result from both the lack of ULT initiation in gout patients by health professionals, but also poor medication adherence (or compliance) by patients [7–9].

A recent systematic review and meta-analysis by Scheepers et al. 2017 shows a large variation in adherence to ULT across studies, with 12-month adherence rates ranging from 3 to 78%, and non-persistence rates ranging from 54 to 87% after 1 year, in prescription and claims-based studies [10]. This could partly be explained by the fact that many of these studies were performed using data collected in health care administrative databases that apply to specific subpopulations of gout patients, for example, databases restricted to specific insurance types, or healthcare settings. Moreover, their results show that only three previous studies described adherence to ULT in European gout patients.

We set out to gain insight into the dispensing patterns, as well as the level of compliance and persistence to commonly prescribed ULT drugs for the treatment of hyperuricemia and gout, in a nationally representative study in the Netherlands.

## Materials and methods

### Data source

Data for this nationally representative, retrospective, prescription-based study were obtained from IQVIA’s Real-World Data Longitudinal Prescription database (LRx, Amsterdam, The Netherlands). The database contains anonymous patient prescription records, including patient (e.g., age, gender), dispensing (e.g., pharmacy, prescription date), medication (e.g., name, dose, strength, therapy duration), and prescriber information. For this study, dispensed ULT prescriptions of allopurinol, febuxostat, and benzbromarone prescribed by rheumatologists, general practitioners, and internists were selected. In total, the database provides a coverage of approximately 75% of all prescriptions dispensed in The Netherlands, represented by both retail pharmacies and dispensing general practitioners. Data was available from the time period of 1 November 2013 up till 31 July 2017.

### Measuring compliance and persistence

Reporting standards for retrospective database studies on measuring compliance and persistence were followed in describing the study results [11]. To identify patients newly starting on ULT, patients were selected who did not receive any ULT prescription (allopurinol, febuxostat, or benzbromarone) in the 6 months prior to their first ULT dispensing in the defined study enrollment period, which was between 1 May 2014 and 31 July 2016. Since ULT is also prescribed for conditions other than gout, and the diagnosis of patients was unknown, patients < 18 years old at the time of first ULT dispensing were excluded. Similarly, patients whose baseline prescription was prescribed by a professional other than a general practitioner or rheumatologist, were excluded. However, follow-up prescriptions by internists were included for calculating compliance and persistence. Prescriptions with a dispensed duration of zero days were excluded.

Compliance to ULT was defined as the proportion of days covered (PDC) by medication over a period of 12 consecutive months, correcting for any overlap in prescriptions [11]. Patients were not required to be continuously using ULT (persistent). For comparability with previous studies, the PDC was dichotomized, and patients with PDC ≥ 0.80 were considered compliant [10]. Persistence refers to the extent to which patients continue their medication over 12 months, without exceeding a refill gap of 30 days. The time to discontinuation was determined in days.

For the analyses of compliance and persistence, we made three assumptions; (1) intake of the medication by patients was done in accordance with the issued prescription, in terms of duration and frequency; (2) patients were initiated on life-long ULT for the treatment of gout and hyperuricemia; and (3) dispensed prescriptions were considered to be taken for the first time on the same day of dispensing, except when a patient still had medication available from the previous prescription; in those cases medication intake started the day after the medication of the previous prescription was exhausted. For calculating compliance and persistence, no distinction was made in type of ULT received.

### Statistical analysis

Means and standard deviations, or medians and first and third quartiles were used to summarize general characteristics of the patient population, dispensing patterns, as well as compliance and persistence levels after 1 year. Univariable and multivariable regression analyses were performed to identify factors associated with PDC and time to treatment cessation. Since the PDC variable is naturally bounded between 0 and 1, and had a “U-shaped” distribution with relatively high numbers of patients at the extremes (i.e., 0.10 ≥ PDC ≥ 0.90), we used beta regression analysis, with a logit link function. Overall goodness of fit was examined using the pseudo *R*-square statistic, and statistical significance of the coefficient estimates were tested using partial Wald statistics. This was accomplished by using the betareg R package [12]. Factors associated with time to treatment cessation were studied using cox-regression analysis using IBM SPSS Statistics version 23. For testing the cox proportional hazard assumption, log minus log graphs were visually inspected.

## Results

### Patient characteristics

ULT prescriptions of 130,232 patients were dispensed by rheumatologists, general practitioners, or an internist between November 2013 and July 2017. Of these, 45,654 patients met the selection criteria, of which the majority were male (*n* = 34,761, 76.1%). The mean (SD) age was 65.7 (14.1) years.

### Dispensing patterns

The total number of ULT prescriptions dispensed was 377,309 with the number of ULT prescriptions per patient ranging from 1 to 297, and a median (first, third quartile) of 5 (3, 7). The most common initial ULT prescription dispensed during the study enrollment period was allopurinol (*n* = 44,068, 96.5%), followed by benzbromarone (*n* = 1386, 3.0%) and febuxostat (*n* = 200, 0.4%). Most patients were issued their first ULT prescription by general practitioners (*n* = 37,917, 83.1%). The median (first, third quartile) ULT treatment duration dispensed was 30 (15, 60) for rheumatologists and 30 (15, 90) days for general practitioners, respectively. Table 1 provides an overview of the initial type of ULT and dosages dispensed by both prescribers.

**Table 1:** Initial ULT dispensed among general practitioners and rheumatologists

Type of ULT	General practitioner, % (*N*)^1^	Rheumatologist, % (*N*)^2^
Allopurinol 100 mg	75.9 (28773)	72.1 (5576)
Allopurinol 200 mg	1.3 (476)	0.4 (31)
Allopurinol 300 mg	20.1 (7630)	20.4 (1582)
Febuxostat 80 mg	0.2 (83)	1.5 (113)
Febuxostat 120 mg	0.0 (4)	0.0 (0)
Benzbromarone 100 mg	2.5 (951)	5.6 (435)

*ULT* urate-lowering therapy

^1^Percentage of total group, *N* = 37,917

^2^Percentage of total group, *N* = 7737

### Compliance

PDC values had a median of 0.82 and first and third quartiles of 0.33 and 0.99, respectively. In total, 51.7% (*n* = 23,602) patients were categorized as compliant.

Gender, age, and first prescriber were found to be associated with PDC, according to the Wald statistics (Table 2). In the multivariable beta regression model, the predicted value for PDC was 53%, when all covariates were set to zero. Ceteris paribus the predicted PDC value increased by 8% if a rheumatologist was the first prescriber instead of a general practitioner, and by ~ 3% for each increasing incremental decade of the patient’s age. The predicted value for PDC decreased by 3% for female patients. The pseudo *R* square for the multivariable model was 0.024.

**Table 2:** Variables associated with PDC

	Univariable analyses			Multivariable analyses^1^		
	Gender	Age	Prescriber	Gender	Age	Prescriber
Intercept	0.957	0.252	0.890	0.139	0.139	0.139
β-coefficient	− 0.044*	0.011**	0.336**	− 0.127**	0.012**	0.348**
SE	0.014	< 0.000	0.015	0.014	< 0.000	0.015
Pseudo *R*^2^	< 0.000	0.011	0.011	0.024	0.024	0.024

*SE*, standard error; *PDC*, proportion of days covered

^1^Model: PDC ~ gender + age + prescriber

*P < 0.01; **P < 0.001

### Persistence

The median (first, third quartile) time to discontinuation of ULT was 248 (83, 420) days for the entire population, and after 1 year 42.7% (*N* = 19,509) of the patients were still persistent. In general, major drops in persistence were seen after 30 (*n* = 3827) and 90 (*n* = 4093) days of treatment, which are common durations of prescribed ULT medications. Noticeably, for 50.2% (*n* = 1922) and 38.9% (*n* = 1593) of the patients stopping at day 30 and 90 respectively, only one ULT prescription was dispensed in 1 year time.

**Fig. 1:**
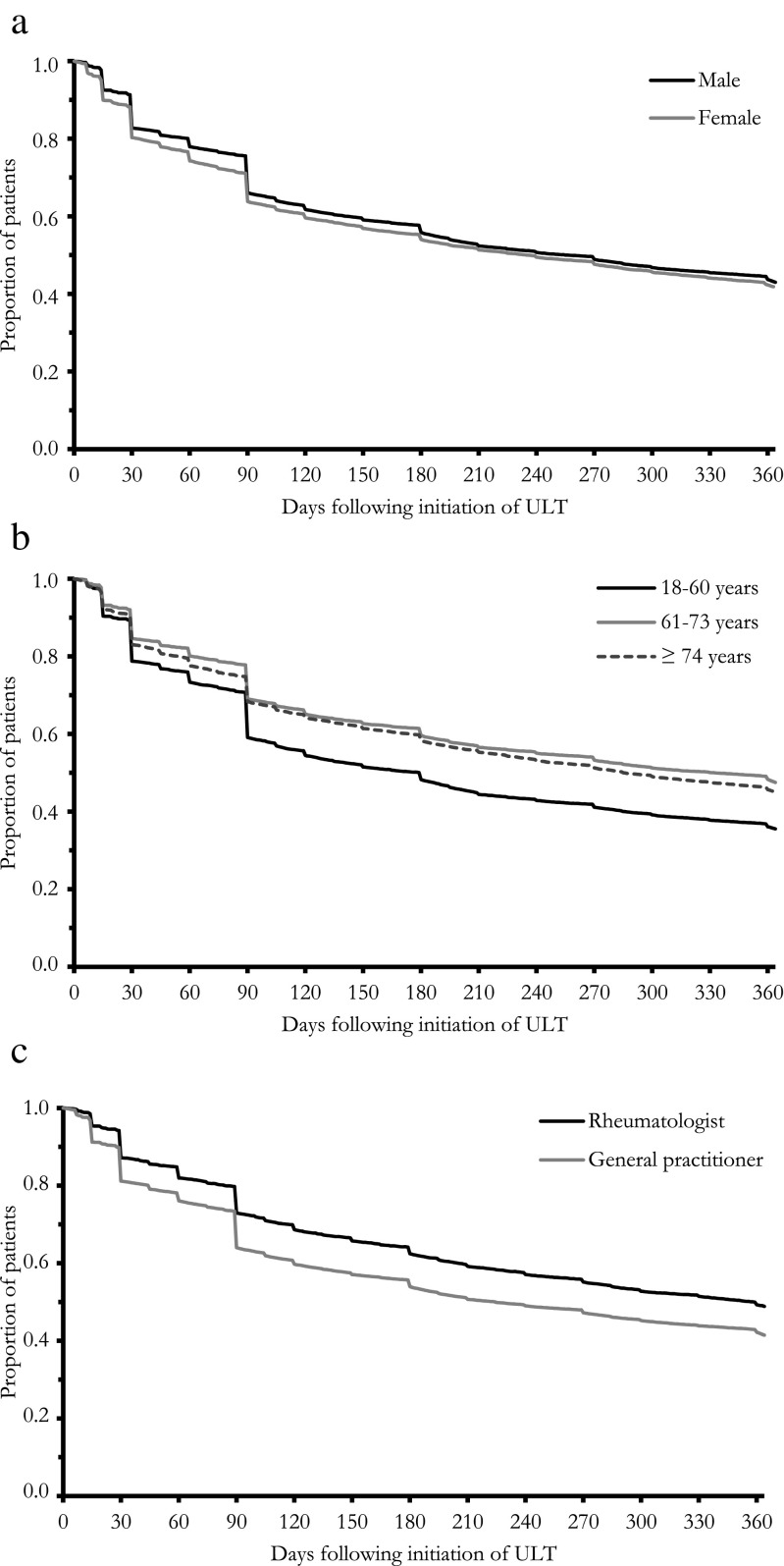
Graphs of the proportion of patients who continued treatment with urate-lowering therapy (ULT) 1 year after initiation of treatment. **a** The level of continuance stratified by gender, **b** stratified by age, and **c** stratified by initial prescriber of ULT

Visual inspection of the log-minus-log hazard function plots for age, gender, and first prescriber supported that the assumption of proportional hazards was met. When applying multivariate cox-regression for gender, age, and initial prescriber of ULT, hazard ratios (HR) differed significantly between groups (Table 3, Fig. 1). Males had a 10.3% (HR 0.897, 95% CI 0.87–0.92) lower probability of treatment cessation at any point in time, compared to women. Also, older patients had a statistically significant lower probability of stopping treatment at any point in time compared to younger patients. Finally, the HR for patients initially treated by a rheumatologists compared to a general practitioner was 0.788 (95% CI, 0.76–0.82), suggesting the probability of discontinuing medication is 21.2% less likely at any point in time for patients initially treated by a rheumatologist.

**Table 3:** Multivariate cox-regression for persistence

	HR	95% CI	Time to discontinuation in days, median (Q1, Q3)	Persistent at 12 months (%)
Gender				
Female^1^	–	–	236 (60, 401)	41.9
Male	0.897*	0.87, 0.92	257 (90, 420)	43.0
Age group				
18–60^1^	–	–	180 (60, 404)	35.6
61–73	0.713*	0.69, 0.73	333 (90, 438)	47.5
≥ 74	0.750*	0.72, 0.77	286 (85, 404)	45.0
Prescriber				
General practitioner^1^	–	–	225 (72, 408)	41.5
Rheumatologist	0.788*	0.76, 0.82	359 (90, 450)	48.9

-, not applicable; *HR*, hazard ratio; *CI*, confidence interval; *Q1*, quartile 1; *Q3*, quartile 3

^1^Comparator group for HR

*Significant difference at *p* < 0.05

## Discussion

This study evaluated the compliance and persistence to commonly prescribed ULT drugs for the treatment of gout and hyperuricemia, as well as factors associated with these, on a national scale.

Based on our results, compliance and persistence to ULT for the treatment of gout and hyperuricemia seems suboptimal in the Netherlands, revealing ULT dispensing patterns that are inconsistent with national and international management guidelines for gout. After 1 year, only 42.7% of the patients were still getting their prescriptions refilled, and 51.7% of the patients had ULT medication coverage of at least 80% of the study days. These findings further substantiate previous reports where suboptimal medication adherence to ULT has been highlighted as a concern in gout patients, in various healthcare systems [6, 13–15]. In fact, an earlier report from Germany and the United Kingdom described similar discouraging results [16]. Although we can only speculate on the reasons for poor adherence, we found that patients whose initial prescriber of ULT was a rheumatologist had better treatment adherence. This suggests that establishing local networks between rheumatologists and general practitioners, as previously suggested in an editorial in this journal, may be helpful for enhancing gout care [17]. Such initiatives, and in particular their influence on medication adherence, should be investigated in The Netherlands.

We found that increasing age, being male, and initiation of ULT by a rheumatologist were factors associated with compliance and continuation of ULT, although it should be noted that these variables explained only a limited amount of the total variance in PDC scores. Nevertheless, the findings that increasing age and male sex were protective factors are consistent with previous research [18]. Moreover, our results show a comparatively strong protective effect for the initial prescriber of ULT with respect to compliance, which is a factor that has thus far been studied in a limited number of studies [19].

An important strength of the study is the large, national coverage provided by the database, and the large number of patient records evaluated. It suggests that the presented results may adequately reflect the adherence levels of Dutch patients to ULT. However, our study also had some limitations. First, considering the definitions we maintained for compliance, the PDC was calculated for each patient over the course of the study year. This means the PDC scores after 1 year were to a large extent determined by the level of persistence of that patient in that same year. Furthermore, our database did not contain information regarding the clinical diagnosis of disease for which ULT was indicated. As such, patients without the diagnosis gout and only indicated for hyperuricemia could have been included, potentially introducing bias to the results. Nevertheless, by applying our patient selection criteria, the probability that the database contained gout patients increased. Lastly, we applied a 0.80 cut-off point for good compliance to facilitate comparison with earlier studies. However, it is currently not known if this cut-off value is clinically relevant for gout patients on ULT. As done for other diseases, we suggest empirical evidence supporting the optimal level of adherence should become available for gout and hyperuricemia, to assist in interpreting compliance levels found among patients on ULT [20].

In conclusion, our results from real-life prescription data show that medication compliance and persistence to ULT for the treatment of gout and hyperuricemia is suboptimal, and not in line with management guidelines for gout recommending life-long ULT. Future studies addressing the reasons for treatment cessation and improving treatment adherence are warranted.
